# Relationship Between the Activities of Gloss-Selective Neurons in the Macaque Inferior Temporal Cortex and the Gloss Discrimination Behavior of the Monkey

**DOI:** 10.1093/texcom/tgab011

**Published:** 2021-02-10

**Authors:** Mika Baba, Akiko Nishio, Hidehiko Komatsu

**Affiliations:** 1 Brain Science Institute, Tamagawa University, Tokyo 194-8610, Japan; 2 National Institute for Physiological Sciences, Aichi 444-8585, Japan

**Keywords:** gloss perception, IT cortex, macaque, microstimulation, muscimol

## Abstract

In the macaque monkey, neurons that selectively respond to specific gloss are present in a restricted region of the central part of the inferior temporal (IT) cortex. Although the population activity of these neurons is known to represent the perceptual gloss space, the involvement of their activity in gloss perception has not been directly tested. In the present study, we examined the causal relationship between the activities of gloss-selective neurons and gloss perception by applying electrical microstimulation or injection of small amounts of muscimol (GABA_A_ agonist) to manipulate neural activities while monkeys performed a gloss discrimination task. We found that microstimulation within or in the vicinity of the region where gloss-selective neurons were recorded induced bias toward higher gloss judgment. With muscimol injection, gloss discrimination performance was degraded in one monkey after the first injection into the region where gloss-selective neurons were recorded. These results suggest that gloss discrimination behavior is mediated by the activities of a gloss-selective network that includes the gloss-selective region in the central IT cortex examined here.

## Introduction

Glossiness is an important visual attribute that provides us with information about the material and surface condition of objects. Although numerous psychophysical studies of glossiness perception have been conducted (Ferwerda et al. 2001; Fleming et al. 2003; Motoyoshi et al. 2007; Marlow et al. 2011; Motoyoshi and Matoba 2012), the underlying neural mechanisms are not yet well understood. Recently, brain regions differentially activated by stimuli with various levels of glossiness were examined in electrophysiological and imaging studies of nonhuman primates and humans. In both macaques and humans, regions strongly responding to glossy visual stimuli were found in the inferior temporal (IT) cortex and the equivalent ventral higher visual cortex (Nishio et al. 2012; Okazawa et al. 2012; Wada et al. 2014; Sun et al. 2015; Miyakawa et al. 2017). Electrophysiological experiments examining the activities of neurons in the IT cortex of the monkey revealed that gloss-selective neurons were localized in a region of the lower bank of the superior temporal sulcus (STS) in the central IT (CIT) cortex. Moreover, a population analysis of the activities of these gloss-selective neurons indicates that they precisely express the perceptual parameters of glossiness important for gloss perception (Nishio et al. 2014). From these results, it is expected that the activities of gloss-selective neurons in the monkey IT cortex are relevant to gloss perception; however, no study has examined the causal relationship between the responses of these neurons and gloss perception.

In various regions of the visual cortex, electrical microstimulation and muscimol injection have been used to investigate the causal relationship between the activities of stimulus-selective neurons and corresponding perception (Salzman et al. 1990; DeAngelis et al. 1998; Chowdhry and DeAngelis 2008). With these techniques, the activities of stimulus-selective neurons around the electrode tip or injection site can be manipulated: electrical microstimulation will activate neurons in the vicinity of the electrode tip, while injection of muscimol will inhibit neural activities in the vicinity of the injection site. These techniques have been applied in the macaque IT cortex to manipulate the activities of neurons sensitive to various visual attributes, including faces (Afraz et al. 2006; Moeller et al. 2017; Sadagopan et al. 2017), three-dimensional shapes (Verhoef et al. 2012), coarse orientation (Adab and Vogels 2016), and object images (Rajalingham and DiCarlo 2019). In these studies, behavioral effects were generally consistent with the stimulus selectivity observed at the same locations. We therefore expect these techniques would also be effective for examining the role of gloss-selective neurons in gloss discrimination behavior.

In the present study, we used the aforementioned techniques to test whether perceptual gloss judgment is affected by manipulating the activities of gloss-selective neurons in the macaque CIT cortex. We first identified the region where gloss-selective neurons are localized. Then, while the monkeys were performing a gloss discrimination task, electrical microstimulation or muscimol injection was applied to investigate how behavioral performance of the gloss discrimination task was affected by the manipulations. We found that microstimulation at the site where gloss-selective activities were recorded or at slightly anterior sites induced bias toward higher gloss judgment. For muscimol injection, gloss discrimination performance was degraded after the first injection into this region in one monkey. We suggest that this region works as part of a neuronal network responsible for gloss discrimination behavior.

## Materials and Methods

### Overview of Experimental Procedure and Apparatus

This study consisted of two sets of experiments. In the first set, we mapped the gloss selectivity of neurons in the lower bank of the STS in the CIT cortex and examined the effects of electrical microstimulation. In each daily session, a microelectrode was inserted vertically into the lower bank of the STS, and the gloss selectivity of the multineuronal activity at different penetration depths was tested while the monkeys performed a fixation task. Then, in the same daily session, electrical microstimulation was applied at one depth while the monkeys performed a gloss discrimination task. After completion of the first set of the experiments, we conducted the second set, which entailed muscimol injection at several sites selected based on the results obtained in the first set of the experiments. We examined the effects of muscimol injection on the performance of the gloss discrimination task. Details of the procedures at each stage are described in the following and information on the samples of the present study is summarized in Table 1.

**Table 1:** Summary of the microstimulation experiment and the muscimol injection experiment in each monkey

#	Monkey G	Monkey T
**Electrical microstimulation experiment**		
Coordinate	19	13
Electrode penetration	34	25
Recorded unit	141	129
Gloss-selective unit	6 (gloss:5, matte:1)	10 (gloss:9, matte:1)
Microstimulation	28	25
**Muscimol injection experiment**		
Coordinate	3	5
Injection	4	7

Notes: (Top panel) Summary of the microstimulation experiment. Numbers of coordinates to which microelectrodes were penetrated for the stimulation experiment, electrode penetrations, recorded MUAs, gloss-selective MUAs, and microstimulation experiments are shown. (Bottom panel) Summary of the muscimol injection experiment. Numbers of coordinates to which injectrodes were penetrated, and muscimol injection experiments are shown.

We used 2 monkeys (monkey T and G, males; *Macaca fuscata* weighing 6.2–7.4 kg) in this study. One of the monkeys (T) was used in our previous study examining the neural selectivity for gloss (Nishio et al. 2012, 2014). In that study, this monkey was trained only a visual fixation task, and gloss discrimination task was newly trained for the present study. Also, recording hemisphere is different from the previous studies, so there is no overlap in the neural samples between the present and previous studies. Another monkey (G) has not been used previously. During the experiments, each monkey was seated on a primate chair, and faced a CRT monitor (TOTOKU) at a distance of 85 cm. Before starting the experiment, a head holder and a recording chamber (rectangular in shape with an opening 10 x 15 mm at the edge) made of plastic were surgically attached to the skull under aseptic conditions and general anesthesia. The stereotaxic coordinates of the center of each recording chamber were 22 mm lateral and 10–12 mm anterior. Physiological experiments were conducted on one hemisphere in each monkey (right hemisphere of monkey T and left hemisphere of monkey G). During the experiment, the monkey’s eye position was monitored using an infrared eye camera system (ISCAN). After the experiments, the animal was administered an overdose of pentobarbital sodium (Somnopentyl) and perfused for histological examination. All procedures for animal care and experimentation were in accordance with the U.S. National Institutes of Health (NIH) Guide for the Care and Use of Laboratory Animals (1996) and were approved by our institutional animal experimentation committee.

### Behavioral Task

The 2 monkeys were trained to perform a visual fixation task and a gloss discrimination task. In the first set of experiments, the gloss selectivity of neural responses was assessed while the monkey under study performed the fixation task; the effects of electrical microstimulation were then assessed while the monkey performed the gloss discrimination task. In the second set of experiments, the effects of muscimol injection were tested while the monkey performed the gloss discrimination task.

In the fixation task, the monkey was first required to keep his eye on a fixation point at the center of the monitor for 500 ms. After the offset of the fixation point, in a single trial, 5 stimulus images appeared in succession for 300 ms each after a 300-ms prestimulus blink period. The stimulus images subtended approximately 5° of visual angle and were presented at the center of the monitor with a gray background (10 cd/m^2^). The monkey was required to maintain fixation on the center of the stimulus image during the entire period of a trial to obtain a drop of liquid reward.

**Figure 1:**
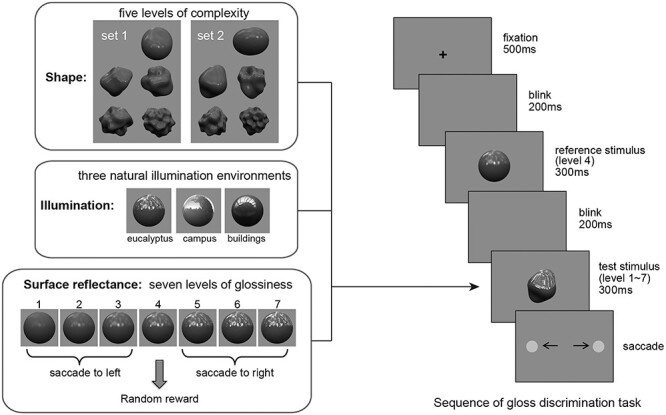
Overview of the visual stimuli used for the gloss discrimination task (Left). Five different shapes, 3 different illuminations, and 7 different surface reflectances (7 levels of glossiness) were used to generate the various glossy objects using computer graphics. Each single test stimulus was rendered using a particular combination of these 3 elements. A shape set consisting of 5 different levels of complexity was systematically generated using an algorithm based on spherical harmonics, and a novel shape set was used for each daily session. Two examples of shape sets are shown as set 1 and set 2. Overview of the gloss discrimination task sequence (Right). After a 500-ms fixation period, a reference stimulus appeared (a sphere exhibiting the middle level glossiness). After a subsequent 200-ms blink period, the test stimulus exhibiting 1 of 7 different levels of glossiness appeared for 300 ms. Two gray circles (targets) appeared to the right and left at the offset of the test stimulus. Animals were trained to make a saccade to either the left or right target to answer whether the test stimulus was more glossy or less glossy than the reference stimulus to get reward. For the middle level of glossiness (level 4), the monkey was randomly rewarded regardless of the saccade direction.

In the gloss discrimination task, the monkey was required to judge the glossiness of various object images, each of which exhibited 1 of 7 levels of glossiness. An overview of the time sequence of the gloss discrimination task is shown in Figure 1, right. In each trial, the monkey was required to fixate on a point at the center of the monitor for 500 ms at the start of the task. The fixation was followed by a 200-ms blink period, after which a reference stimulus appeared for 300 ms. The reference stimulus was a spherical object with the middle level glossiness (level 4) (details of the glossiness level in the task are explained in “Visual Stimuli” section). After the subsequent 200-ms blink period, a test stimulus exhibiting 1 of the 7 glossiness levels appeared for 300 ms. Following the offset of the test stimulus, 2 gray circles (targets) appeared, and the monkey was required to make a leftward or rightward saccade to answer whether the test stimulus was less glossy or more glossy than the reference stimulus. When the test stimulus had middle level of glossiness (level 4), which was the same level as the reference stimulus, the monkey was randomly rewarded regardless of the direction of the saccade. Correct directions of the saccade were counterbalanced between monkeys. There were 210 conditions in the task, which consisted of 105 test stimuli (7 glossiness levels × 5 shapes × 3 illuminations) and 2 stimulation conditions (with and without stimulation). Each of those 210 conditions was tested once during the recording at one site. We used a novel set of 5 object shapes in each daily session. This prevented the monkeys from using the pattern of highlights or shadings to perform discrimination of object stimuli because those patterns changed every day. For the training of the gloss discrimination task, it took about 9 months (monkey T) or 8 months (monkey G).

### Visual Stimuli

We used Radiance software (http://radsite.lbl.gov/radiance/) to render realistic object images with variously glossy appearances. The stimulus set used for the gloss discrimination task consisted of combinations of object shapes with 5 levels of complexity, 3 different illuminations (Eucalyptus, Campus, and Building, selected from the Debevec high dynamic range dataset; http://ict.debevec.org/), and 7 levels of glossiness, resulting in 105 different images (see Fig. 1, left and Supplementary Fig. 1). The 5 levels of complexity ranged from a smooth ellipsoid-like shape to complex bumpy shapes, which were parametrically generated using an algorithm based on a spherical harmonics function (Shimokawa et al. 2019). In each daily session, a different set of shapes was generated by using a different set of random numbers corresponding to each dimension of spherical harmonics function generated by rand function of MATLAB (Fig. 1, left). To obtain different set of random numbers, we incremented the value of seed for random number generation in each daily session. We made 7 levels of glossiness systematically by choosing parameters of surface reflectance at equal intervals along the diagonal axis in the “*c*-*d* space” (Supplementary Fig. 2; Ferwerda et al. 2001; Nishio et al. 2014). The “*c*” and “*d*” represent 2 principal axes of perceptual gloss space, and those parameters were derived from 3 physical parameters of surface reflection: specular reflectance (ρs), which indicates the strength of the specular reflection; diffuse reflectance (ρd), which indicates the strength of the diffuse reflection; and the spread of the specular reflection (α), which is caused by fine scale unevenness or roughness of the surface (Ferwerda et al. 2001). It is thought that “*c*” is related to the contrast of the highlights and “*d*” is related to the sharpness of the highlights. The range of the glossiness, which determined the task difficulty, was adjusted for each monkey: the range of parameter “*c*” was 0.029–0.119 for monkey T and 0.0215–0.1265 for monkey G, and the values of parameter “*d*” were determined automatically so as to preserve the Δ*c*/Δ*d* ratio constant as 1.78 and achieve a perceptually uniform *c*-*d* space (Ferwerda et al. 2001). While the illumination used for rendering the reference stimulus was constant (Eucalyptus) for monkey T, it was the same as the test stimulus in each trial for monkey G whose performance was less stable. Because of the large variation of shapes of the test stimulus, gloss discrimination task cannot be reduced to contrast discrimination task even when the illumination of the reference stimulus was constant (see Supplementary Fig. 6).

The stimulus set for testing neural selectivity for gloss consisted of 5 different shapes and 7 different glossiness levels in a single illumination condition (Eucalyptus), giving 35 different images (Supplementary Fig. 3). To test whether the neural activity was selective for the glossiness level or just for the mean luminance level, a set of “shuffled images” in which the pixels were randomly rearranged inside the contour of the objects was also used (Supplementary Fig. 3).

### Mapping Gloss Selectivity and Electrical Microstimulation

In each daily sessions, we inserted a tungsten microelectrode (FHC) at selected coordinates through a stainless steel guide tube fitted into a hole in a plastic grid attached to the recording chamber. The grid had many holes lining up at 1-mm intervals in the anterior–posterior and medial–lateral directions. Prior to the electrical microstimulation, we tested the neural selectivity to glossiness of the target cortical area by recording multiunit activity (MUA). When the electrode tip entered the lower bank of the STS, we tested the gloss selectivity of the MUA at 100- to 200-μm intervals. We applied a criterion to the MUAs in which a threshold was set so that the baseline activity computed during a 300-ms period before the onset of the stimulus would be 40 spk/s. To test the gloss selectivity of the MUAs, we presented 35 object images (5 shapes × 7 glossiness levels) and 7 shuffled images of the optimal shape, which was determined as the shape yielding the largest response. In each daily session, after MUAs were recorded at about 5 different depths, an electrical microstimulation experiment was conducted using the same electrode. The electrode was drawn back to the center position among all the sites at which gloss-selective responses were recorded or to the center position among all the sites examined at which no gloss-selective MUA was recorded. While the monkey under study performed the gloss discrimination task, biphasic electrical pulses (35 μA, 200 Hz, 200 ms pulse width, positive–negative) were applied for 300 ms, starting 50 ms after the onset of the test stimulus and ending 50 ms after the offset of the test stimulus. In half of the trials, microstimulation was applied, while in the remaining half, no stimulation was applied. Usually, electrodes were inserted at the same surface coordinates twice in different daily sessions in order to conduct neural recordings and electrical microstimulation at different depths and cover the entire depth of the lower bank of the STS. In total, 25 penetrations at 13 coordinates were made for monkey T, and 34 penetrations at 19 coordinates were made for monkey G. Microstimulation experiments were conducted for all those penetrations with monkey T (*n* = 25), while 28 microstimulation experiments were conducted with monkey G. The remaining 6 penetrations in monkey G were made at the beginning of the experiments, and only the test for neural selectivity was made.

### Muscimol Injection

After the selectivity mapping and electrical microstimulation experiments were completed, reversible inactivation of the target area was achieved by injection of the GABA_A_ agonist muscimol. Muscimol was injected using a custom-made microinjectrode, which consisted of a fused silica tube (Polymicro, i.d. = 75 μm, o.d. = 150 μm) attached to a Hamilton Syringe and a fine tungsten microelectrode (Frederick Haer). Both the silica tube and the electrode were contained within a polyimide tube (MicroLumen, i.d. = 410 μm, o.d. = 457 μm) such that both were inserted into the brain as a single bundle (Kliem and Wichmann 2004). The tip of the electrode was positioned 0.5 mm below the tip of the silica tube through which muscimol was injected, and we were able to precisely monitor the neural activity around the tip of the injectrode. In each experiment, a stainless guide tube was initially inserted into the cortex slightly above the STS. Then, the microinjectrode was inserted into the target cortex through the guide tube at the same coordinates based on the depth record in the preceding recording experiment. After neural activity was confirmed, 2 μL of muscimol (10 mg/mL concentration) were manually injected at rate of 0.1 μL/min. In some later part of experiments (3 out of 11 cases), larger amount of muscimol was injected (3 μL in 1 case or 4 μL in 2 cases). Performance of the gloss discrimination task was tested before the muscimol injection and 30 min and 18 h after the muscimol injection. We chose 18 h based on a previous study (Chowdhury and DeAngelis 2008) that showed the largest effects of muscimol injection into the extrastriate cortex were observed at that time. The effects of muscimol injection were analyzed by comparing the performance before and after the injection (see below). When an effect of muscimol injection was observed 18 h after injection, task performance was also recorded 42 h after the injection to confirm recovery.

### Data Analysis

In the neural recording experiment to test gloss selectivity using the visual fixation task, neural responses were analyzed only for correct trials. The minimum number of repetitions of each stimulus accepted for analysis was 5. Visual responses were computed as follows. First, mean neural activity was computed for a 300-ms period beginning 50 ms after stimulus onset. To compute the neural response to each stimulus, baseline activity was subtracted. To examine the significance of neural response tuning to gloss, we applied ANOVA where the modulation of firing rates dependent on the difference in glossiness level was tested (*P* < 0.05). When a significant modulation to gloss was confirmed with the optimal shape and at least one other shape, Pearson’s correlation coefficient was computed between the tuning to the optimal shape and that to the other shapes. When the response modulation to the shuffled images was also significant, the correlation coefficient between the optimal shape and the shuffled images was also computed. A neuron was classified as gloss-selective when there was significant correlation between the tunings to the optimal and other shapes and there was no significant modulation to the shuffled images or no significant correlation between the tunings to the optimal shape and shuffled images.

To evaluate the behavioral performance of each monkey in the gloss discrimination task, we constructed a psychometric function, examples of which are shown in Fig. 3*B*,*D*. The proportion of trials in which the monkey chose the target that corresponded to the test stimulus being glossier than the reference stimulus (gloss choice) was plotted for each of the 7 levels of glossiness. All the data obtained using different shapes and illuminations were averaged for each glossiness level. Then, to obtain the psychometric function, the data were fitted by a logistic function using the following equation

$$y=\frac{1}{1+{e}^{-a\left(x-b\right)}}$$

where *x* is the glossiness level (1–7), *a* corresponds to the slope of the function that represents the sensitivity to the difference in the glossiness, and *b* represents the offset that yields a 50% gloss choice. One dataset from monkey G was removed from further analysis because the fitting error for the function was extremely large. To quantify the change in behavioral performance elicited by electrical microstimulation, we computed the difference in parameters *a* and *b* between the conditions in which electrical stimulation was applied and those where it was not applied. A random permutation test was then applied to assess whether the difference was statistically significant, as follows. For every glossiness level, there were 30 samples of behavioral data (3 illuminations × 5 shapes = 15 in both the stimulation and no-stimulation conditions). To perform the permutation, we generated two datasets by randomly selecting 15 samples from among the original 30 samples and generating a novel psychometric function for each of the 2 datasets. We then computed the differences in slope and offset between the 2 psychometric functions. By repeating this procedure 10 000 times, we generated a distribution of Δ*a* and Δ*b*. When the original value of the parameter difference was within 2.5% of the maximum or minimum range of the distribution, the difference was regarded significant.

A similar procedure was used for analysis of the behavioral performance after muscimol injection. In that case, the 2 conditions were “before drug injection” and “after drug injection.”

## Results

To study the relationship between the activities of gloss-selective neurons and gloss perception, we assessed the effects of electrical microstimulation and muscimol injection while the 2 monkeys performed a gloss discrimination task.

### Mapping of Gloss Selectivity

We first mapped the gloss selectivity of neurons in the lower bank of the STS. Recordings were made at 32 sets of coordinates (13 for monkey T, 19 for monkey G) at 1-mm intervals in the anterior–posterior and medial–lateral directions. We examined the gloss selectivity of an MUA as follows. First, we assessed whether the neural activity was significantly modulated with respect to the 7 levels of glossiness for at least 2 different shapes (ANOVA, *P* < 0.05). The MUA was classified as gloss-selective when there was a significant correlation between the tunings to the optimal and other shapes, and there was no significant modulation in response to shuffled images or no significant correlation between the tunings to the optimal shape and shuffled images. (For more details, see the Materials and Methods).

**Figure 2:**
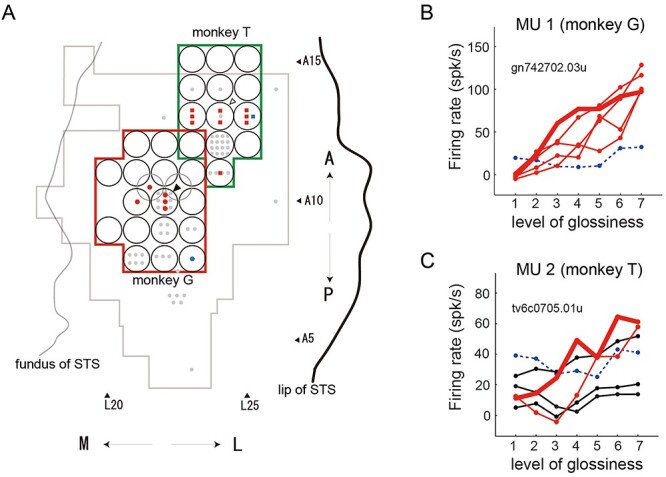
(*A*) The recorded areas in the 2 monkeys are overlaid with the top view of the lower bank of the STS in the right hemisphere (copied from a previous study; Nishio et al. 2012). Recorded sites are enclosed by green contour (monkey T, right hemisphere) and red contour (monkey G, left hemisphere flipped around AP axis). Black open circles indicate the positions of the grid holes for electrode penetrations, and red or blue symbols indicate the positions at which gloss-selective units were recorded. The number of symbols in a single circle indicates the number of gloss-selective units recorded at that locus. Red symbols indicate that the recorded unit responded more strongly to the glossier stimulus, while blue symbols indicate that the response was stronger for the less glossy stimulus. Different symbols represent the different monkeys. Gray contour and gray symbols, respectively, indicate the recoding area and the positions at which gloss-selective neurons were recorded in a previous study. (*B*) Responses to the test stimulus of a representative gloss-selective unit in monkey G. The horizontal axis indicates the level of glossiness of the test stimulus, while vertical axis indicates firing rate. Thick and thin lines represent the neural responses to the optimal shape and to 4 other shapes, respectively. Thin red lines indicate significant variation in responses across the 7 levels of glossiness (ANOVA, *P* < 0.05) as well as significant correlation with the responses to the optimal shape. The blue dashed line indicates the response to the shuffled images. (*C*) Responses of an example gloss-selective unit of monkey T. The format is the same as in *B*. Thin black lines indicate that the responses to a given shape were either not significant or not correlated with the responses to the optimal shape.

The results of the mapping of the gloss-selective neural activities are shown in Figure 2, where each gloss-selective MUA is indicated by a colored symbol (Fig. 2*A*) at the coordinates (black circle) where the MUA was recorded. If multiple gloss-selective MUAs were recorded at different depths along the same electrode penetration, or in a different recording session targeting the same coordinates, a different symbol is depicted for each MUA. In 4 out of 8 coordinates where gloss-selective neurons were recorded, those neurons were recorded at multiple depths in the same electrode penetration suggesting clustering across recording depth (Fig. 2*A*). All gloss-selective MUAs exhibited a roughly monotonic increase or decrease in their response to a change in glossiness (Supplementary Fig. 4). Red symbols in Figure 2*A* represent MUAs that showed stronger responses to glossier stimuli (*n* = 14), while blue symbols represent those that showed stronger responses to less glossy stimuli (*n* = 2). Examples of a gloss-selective MUA recorded from each monkey (MU1 from monkey G and MU2 from monkey T) are depicted in Figure 2*B*,*C**.* MU1 (Fig. 2*B*) showed similar gloss-selective responses to all 5 different shapes. By contrast, MU2 (Fig. 2*C*) showed gloss-selective responses to only 2 shapes (red lines); no significant selectivity was observed for the other 3 shapes (black lines). In both cases, no significant modulation was observed for the shuffled stimuli (blue dashed line). As can be seen in the map in Figure 2*A*, most gloss-selective MUAs responded strongly to higher gloss stimuli, tended to be localized in small areas within each hemisphere and were not uniformly distributed. This result corresponds well with our previous report (Nishio et al. 2012). In Figure 2*A*, the coordinates where gloss-selective neurons were recorded in previous papers are also indicated by light gray symbols on the map. The distribution of the gloss-selective MUAs recorded in the present study clearly overlaps those from the earlier study. As can be seen in this figure, gloss-selective neurons were recorded in the region in the posterior bank of STS whose extent is about 6 mm in AP direction × 5 mm in LM direction in 5 hemispheres including 2 hemispheres examined in the present study. In each hemisphere, gloss selective neurons were mainly recorded in a small area ranging 2–3 mm (gloss-selective region). The position of this gloss-selective region varied from hemisphere to hemisphere within the 6 mm AP × 5 mm LM range.

### Electrical Microstimulation

**Figure 3:**
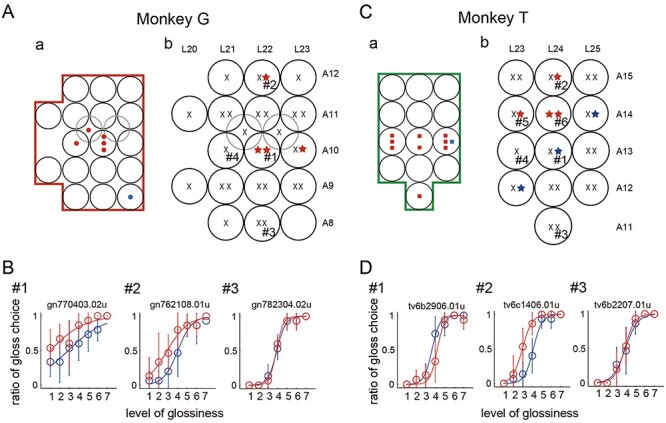
Effects of electrical microstimulation in monkey G (*A* and *B*) and monkey T (*C* and *D*). (*A*,*a*) Results of the mapping of gloss-selective units (same format as in Fig. 2*A*). (*b*) Results of microstimulation tested at each site whose stereotaxic coordinates are indicated. Stars represent significant behavioral changes (horizontal shift of the psychometric function) induced by the stimulation. Red stars indicate bias toward judging the test stimulus to be glossier than the reference stimulus; blue stars indicate bias toward judging the test stimulus to be less glossy. Crosses indicate no significant effect. Multiple symbols at the same site indicate that the stimulation was performed multiple times at different depths. Behavioral results at the sites labeled with # number are shown in *B*. (*B*) Examples of behavioral results obtained at 3 sites whose positions are shown in *A*. The proportion of judgments in which the monkey chose the test stimulus to be glossier than the reference stimulus is plotted for each level of glossiness of the test stimulus. Colors represent with (red) and without (blue) microstimulation, and data points were fitted with a logistic function (curved line). Error bars indicate the standard deviation. (*C* and *D*) Results obtained from monkey T are shown in the same format as in *A* and *B*.

**Figure 4:**
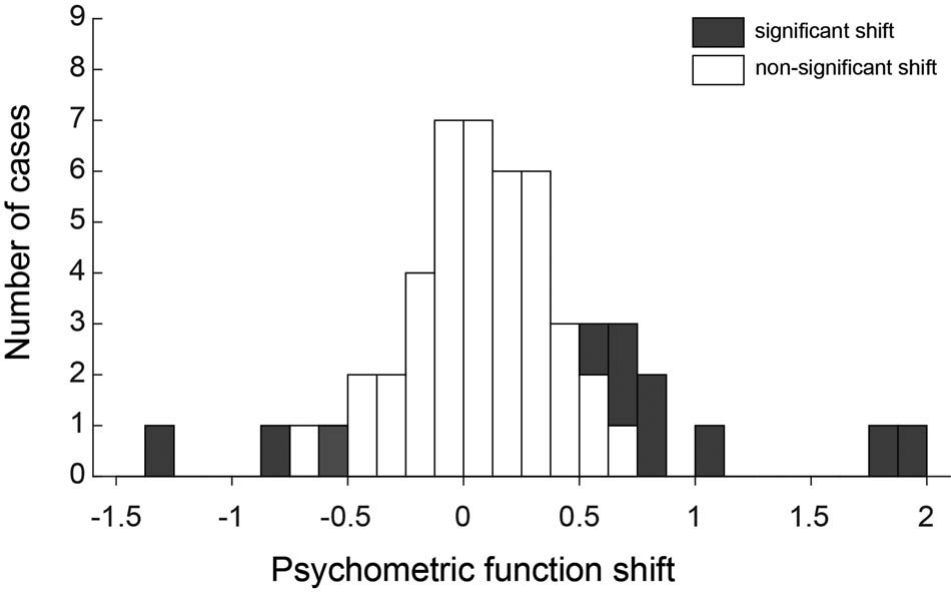
Summary of the horizontal shift of psychometric function in all the microstimulation experiments. Gray bar represents significant effect (*P* < 0.05, permutation test), and open bar represents nonsignificant effect.

At the end of each mapping session, we conducted an electrical microstimulation experiment using the same electrode. The electrode was drawn back to the center position among all the sites where gloss-selective MUAs were recorded or to the center position among all the sites where no gloss-selective MUA was recorded, and the effects of electrical microstimulation were tested. While the monkey performed the gloss discrimination task, we applied electrical microstimulation (35 μA, 200 Hz, 300-ms duration) during the period of test stimulus presentation in half of the trials. Stimulation was applied at one location during a single penetration. Because we carried out 1 or 2 penetrations at the same set of coordinates, electrical microstimulation was conducted once or twice at those coordinates. In total, we conducted 53 microstimulation sessions (25 times for monkey T, 28 times for monkey G) at 32 coordinates where penetrations had been made. Each session consisted of 210 trials (for more details, see the Materials and Methods). Figure 3*A*,*b*,*C*,*b* shows the results of these experiments. Star-shaped symbols indicate that a significant behavioral change was elicited by electrical microstimulation. Nearly all of these changes were manifested as a horizontal shift in the psychometric function, and in one case, the slope of the function was also significantly changed. Figure 4 summarizes horizontal shifts of psychometric function across 53 microstimulation sessions tested. A significant horizontal shift was observed in 11 experiments (4 for monkey G, 7 for monkey T) at 9 locations (3 for monkey G, 6 for monkey T). In 8 of these cases (4 for monkey G, 4 for monkey T), behavioral bias was induced in that the monkey judged the test stimulus to be glossier in trials with electrical stimulation (red star symbols). Three examples of such cases are shown in Figure 3*B* (#1, #2) and 3D (#2) as overlaid psychometric functions with and without electrical stimulation (red and blue, respectively). In the remaining 3 cases (all in monkey T), electrical stimulation induced behavioral bias such that the monkey judged the test stimulus to be less glossy (blue star symbols in Fig. 3*C*,*b*). Psychometric functions illustrating an example of such cases are shown in Figure 3*D* (#1). Cases in which no significant behavioral change was observed are represented by black crosses on the map (Fig. 3*A*,*b*,*C*,*b*), and psychometric functions illustrating two examples of such cases are shown in Figure 3*B* (#3) and Figure 3*D* (#3).

The results described above indicate that electrical microstimulation applied to a region in the posterior bank of the STS in the CIT cortex, where gloss-selective neurons are observed, induces behavioral changes in gloss discrimination behavior. However, the localization of the gloss-selective neurons and the behavioral effects of their stimulation did not correspond precisely. When we compared the map of gloss-selective MUAs (Fig. 3*A*,*a*,*C*,*a*) and that of electrical microstimulation (Fig. 3*A*,*b*,*C*,*b*), at one location both gloss-selective MUAs and a significant behavioral change with stimulation were observed (#1 in monkey G). On the other hand, at other locations neural selectivity and behavioral changes did not completely correspond, and their positions were slightly separated. In some of these cases, we recorded a gloss-selective MUA that responded strongly to higher gloss stimuli, but no significant effect was induced by electrical stimulation (e.g., #4 in monkey G or #4 in monkey T). In another case, we recorded a gloss-selective MUA preferring higher gloss stimuli, but electrical stimulation induced behavioral bias such that the monkey judged the test stimulus to be less glossy (#1 in monkey T). When electrical stimulation led the monkey judge the test stimulus to be more glossy than the reference stimulus, there was a tendency for that behavioral bias to be induced at coordinates slightly anterior to those where gloss-selective MUAs preferring higher gloss stimuli were recorded (#2 in monkey G, #2, #5, and #6 in monkey T). We will consider the relationship between the two maps and the possible causes of this discrepancy in the Discussion.

### Muscimol Injection

**Figure 5:**
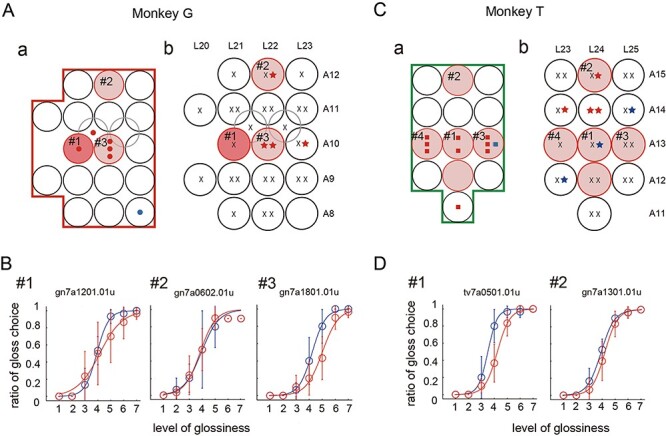
Effects of muscimol injection in monkey G (*A* and *B*) and monkey T (*C* and *D*). (*A*) Results of the mapping of gloss-selective units (*a*) and the microstimulation experiment (*b*) (same format as in Fig. 3*A*); sites where muscimol was injected are shown in pink (dark pink: site with significant effect, pale pink: site with nonsignificant effect) Behavioral results at the sites labeled with #1, #2, and #3 are shown in *B*; those at label #1 and #3 are also referred to in Figure 6. (*B*) Examples of behavioral results obtained at 3 sites whose positions are shown in *A*. The proportion of judgments in which the monkey chose the test stimulus to be glossier than the reference stimulus is plotted for each level of glossiness of the test stimulus. Colors represent behavioral performance obtained before the muscimol injection (blue) and 18 h after the injection (red), and data points are fitted with a logistic function (curved line). Error bars shows the standard deviation. (*C* and *D*) Results obtained from monkey T are shown in the same format as in *A* and *B*.

After the mapping of gloss-selective MUAs and electrical microstimulation were completed, we conducted reversible inactivation experiments. We injected the GABA_A_ agonist muscimol at several coordinates in the recorded region and examined the behavioral effects on performance of the gloss discrimination task. In Figure 5*A,C*, coordinates of muscimol injection are compared with the map of the recordings of gloss-selective MUAs (Fig. 5*A*,*a*,*C*,*a*) or with the map of the electrical stimulation experiments (Fig. 5*A*,*b,C*,*b*). In each panel in Figure 5*A,C*, coordinates where muscimol injections were made are indicated by filled pink circles. Muscimol was injected at 8 coordinates (3 in monkey G, 5 in monkey T) in total. Those coordinates included sites where the gloss-selective MUAs were recorded (e.g., #1, #3 in monkey G, #1, #3, #4 in monkey T in Fig. 5*A,C*) and those where no gloss-selective MUA was recorded (e.g., #2 in monkey G and #2 in monkey T). Each day, muscimol was injected at a single coordinate. At several coordinates, we conducted injection experiments multiple times on different days (2 times for #3 in monkey G, 3 times for #1 in monkey T), so the total number of injection experiments was 11. Injection experiments at the same coordinates were separated by at least 7 days.

**Figure 6:**
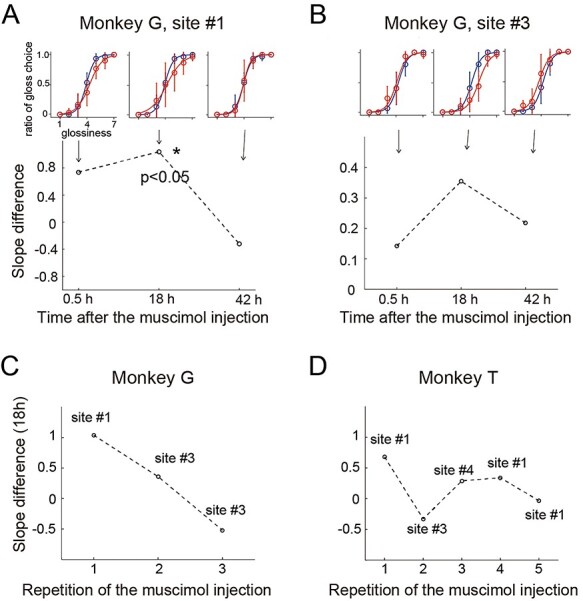
Time-dependent changes in the effects of muscimol (*A* and *B*) and the effects of repeated injections (*C* and *D*). (*A*) Temporal profile of the effects of muscimol injection at site #1 (Fig. 5*A*) in monkey G. Psychometric functions at 3 time points (0.5, 18, and 42 h after muscimol injection, red curves) are compared with that before muscimol injection (blue curves). Below psychometric functions, the difference in the slope of the psychometric functions before and after the drug injection is plotted as a function of time after muscimol injection. The asterisk indicates that the difference was statistically significant (random permutation test, *P* < 0.05). (*B*) Temporal profile of the effects of muscimol injection at site #3 (Fig. 5*A*) in monkey G. The format is the same as in *A*. (*C*) Effects of muscimol injection plotted against the number of muscimol injections at the sites where gloss-selective units were recorded in monkey G. The numbers on the horizontal axis indicate the 1st injection, 2nd injection, and so on. The site numbers indicated above each data point correspond to those in Figure 5*A*. (*D*) Effects of muscimol injection plotted against the number of muscimol injections at the sites where gloss-selective units were recorded in monkey T. The site numbers indicated above or below each data point correspond to those in Figure 5*C*. The format is the same as in *C*.

We observed both slope changes and horizontal shifts in the psychometric functions after muscimol injection (Supplementary Fig. 5). However, the inhibitory effects of the drug were sustained throughout the trial, and it may have affected the perception of both the reference and test stimuli (or had no impact), which makes interpretation of a horizontal/vertical shift in the psychometric function difficult. Consequently, we will consider only slope changes that appeared in the results of the muscimol experiment. A possible prediction of the results of the suppression of the activities of gloss-selective neurons that discriminate different levels of glossiness is a reduction of sensitivity for the discrimination of glossiness. We expected this will appear as the reduction of the slope of the psychometric function. Examples of the effects of muscimol injection in 5 experiments are shown as overlaid psychometric functions in Figure 5*B,D*, where behavioral performance before injection (blue) is compared with that 18 h after the injection (red). In an experiment depicted in Figure 5*B* (#1), a muscimol injection made at coordinate #1 in Figure 5*A*,*a*,*b* in monkey G induced a significant reduction in the slope of the psychometric function. This is the only significant effect in 11 injections, and this site is indicated by dark pink in Figure 5*A*,*a*,*b*. In Figure 6*A*, the change in the slope of psychometric function after the muscimol injection is shown at 3 time points (30 min, 18 h, and 42 h). The slope change was quantified as the difference between the slope obtained at a given time point after the injection compared with that obtained before injection. A slope change was observed 30 min after the injection, though it was not significant. The change was maximal 18 h after injection, and it had disappeared by 42 h after injection. The time course of this result is consistent with a previous report showing that the effect of muscimol injection into the monkey visual cortex was maximum 18 h after injection (Chowdhury and DeAngelis 2008). The effect of muscimol followed a similar time course at coordinate #3 in Figure 5*A*,*a*,*b* (monkey G), where the slope difference was maximum 18 h after injection (Fig. 6*B*), though the slope change was not statistically significant. A significant slope change was observed only once (Fig. 5*B* #1) in a total of 11 experiments. Notably, this result was obtained in the first of 3 experiments in which muscimol was injected at a coordinate where a gloss-selective MUA was recorded in monkey G (#1 and #3 in Fig. 5*A*). Figure 6*C,D* summarizes the results of all the experiments of muscimol injection to the coordinates where gloss-selective MUAs were recorded. In monkey G (Fig. 6*C*), the first injection (depicted in Fig. 5*B* #1) induced a significant reduction in sensitivity, but the effect of the second injection was diminished, and no reduction in sensitivity, or even a slight increase (not significant), occurred with the third injection. Likewise, in monkey T (Fig. 6*D*), the greatest reduction in sensitivity was observed after the first muscimol injection, though the reduction was not significant. This common tendency observed in 2 monkeys provides clues when considering how muscimol affects the sensitivity of gloss judgment behavior, as we will discuss in the Discussion. When muscimol was injected at a site apart from the area where gloss-selective responses were recorded, there was no effect on the performance of the gloss discrimination task in either monkey (#2 in monkey G, Fig. 5*A,B*, #2 in monkey T, Fig. 5*C,D*).

## Discussion

In this study, we investigated the relationship between the activities of gloss-selective neurons in the lower bank of the STS in the CIT and gloss discrimination behavior. To accomplish this, we used electrical microstimulation and muscimol injection to manipulate neural activity while the monkeys performed a gloss discrimination task and examined the effects of the manipulation on task performance. Mapping of neural activities showed that gloss-selective neurons were concentrated in a small region of the lower bank of the STS in both hemispheres examined. This confirms our earlier observation, and now clustering of gloss-selective neurons in this area of the CIT has been observed in 5 brain hemispheres (3 hemispheres in Nishio et al. 2012 and 2 hemispheres in this study). When we applied electrical microstimulation around these regions, a horizontal shift in the psychometric function was induced at some sites, supporting the idea that this region is related to gloss discrimination behavior. However, the distributions of gloss-selective units and the effective sites did not precisely coincide. Finally, a significant reduction in the sensitivity of gloss discrimination behavior occurred after muscimol injection at one site in one monkey. Maximum effects were observed 18 h after injection, and the effect of muscimol was diminished with repeated injections. In the following, we will consider possible causes of these results and discuss how these results can be understood if this region is working as a part of a neuronal network responsible for gloss discrimination behavior.

### How Does Electrical Stimulation Affect Neural Activity?

Electrical microstimulation is considered to be an effective technique for investigating the relationship between visual perception and neural activities that are selective for certain visual attributes, and it has been successfully used to study the extrastriate cortex in macaque monkeys. Those experiments originated from work investigating the relation between the middle temporal area of the visual cortex (MT) and visual motion (Salzman et al. 1990, 1992; Murasugi et al. 1993). In the IT cortex, studies were conducted to investigate the functions of face-selective neurons and neurons selective for three-dimensional shape perception (Afraz et al. 2006; Verhoef et al. 2012). In those experiments, electrical stimulation at the sites where neurons were selective for a given attribute induced a horizontal shift in the psychometric function for the behavioral task to discriminate that attribute, thereby corroborating the causal relationship between the neural activity and the visual perception. In the present study, however, the positions at which the gloss-selective neurons were recorded and the positions at which electrical stimulation induced a behavioral change did not precisely correspond. There are several possible explanations for this discrepancy.

First, one has to consider the possibility that the monkeys performed the task using visual cues irrelevant to gloss perception. For example, a low-level visual feature, such as the luminance or contrast of the image, could potentially change systematically along with the changes in the level of glossiness. If the monkeys relied on these lower-level features to solve the task, it could cause a discrepancy between the localization of the gloss-selective neurons and the effective sites. However, the values of those features would be greatly affected by the illumination environment used to render the stimuli, and our test stimulus set contained a wide range of mean luminance and RMS contrast, even at a single glossiness level. We analyzed the performance predicted if a monkey used only low-level image features as cues to discriminate the 7 levels of glossy images. The resulting performance was much poorer than the actual performance of the monkeys (Supplementary Fig. 6), which makes it unlikely that the monkeys solved the task using only low-level image features. Although skewness is shown to be an important cue for judgment of glossiness (Motoyoshi et al. 2007), it is also indicated that skewness alone cannot explain glossiness perception of object images (Marlow et al. 2011). The present result is consistent with such reports.

Another possible cause for the discrepancy is that electrical microstimulation mainly affected neural activity by stimulating the fibers of neurons. The effects of electrical stimulation on cortical neural activities are complicated, and the precise mechanisms involved are still debated. Some reports argue that close relationships between the stimulus selectivity and the resulting effects on behavior suggest that electrical stimulation activates neurons immediately adjacent to the electrode tip (Histed and Maunsell 2013; Moeller et al. 2017). On the other hand, experimental studies closely examining the effects of electrical stimulation on neural circuits and theoretical analyses showed that electrical stimulation activates neural fibers more effectively than cell bodies (Ranck 1975; Gustafsson and Jankowska 1976; Nowak and Bullier 1998; McIntyre and Grill 2000; Butovas and Schwartz 2003). If neurons with similar stimulus selectivities were densely clustered in a wide region of the cortex, a discrepancy between the location of the stimulation and that of the selective neurons would be obscured by the presence of neurons with similar selectivity. By contrast, the discrepancy would become more obvious if the distribution of selective neurons was less dense and spatially more limited. The former condition is met in the previous studies in area MT and the face patch in the IT cortex, where electrical microstimulation was applied to a region where neurons having selectivity for specific visual attributes were densely clustered across several millimeters (Salzman et al., 1990, 1992; DeAngelis et al. 1998; Afraz et al. 2006). It is also shown that a large fraction of the neurons are selective for three-dimensional shapes in the IT region where electrical stimulation was applied (Verhoef et al. 2012). On the other hand, the latter condition more likely applies to the present study. In our experiment, although gloss-selective neurons were clustered to a certain extent within the cortical region examined, the density of these neurons was not as high as in the studies mentioned above (Table 1). In several penetrations, we could see that gloss-selective neurons were accumulated to some degree, but their distribution (Supplementary Fig. 7) was sparser than the distribution of three-dimensional shape-selective neurons reported by Verhoef (Supplementary Fig. 3 of Verhoef et al. 2012). We speculate that the relatively sparse distribution of gloss-selective neurons as well as the small extent of the region where these neurons are located can explain the discrepancy between the distribution of gloss-selective neurons and the location of the effective sites of electrical microstimulation. When we injected muscimol into sites apart from the coordinates where gloss-selective neurons were recorded, no behavioral change was induced, even when the electrical microstimulation at the same coordinate affected the behavior (#2 in Fig. 5*A*,*C*). This observation is also consistent with the idea that electrical microstimulation mainly activates the neural fibers.

### Possible Cause of the Anterior Bias of the Stimulation Effect

In the present study, the behavioral effect of electrical microstimulation led the monkey to more frequently choose the test stimulus as glossier than the reference stimulus. Notably, the effective sites of electrical microstimulation tended to be slightly anterior to the coordinates where gloss-selective MUAs were recorded. We speculate that one possible cause of this anterior bias is the presence of a neuronal network related to gloss within the IT cortex and that a node of that network located more anterior to the area studied in the present study is closely related to gloss discrimination behavior. It has been shown that there are multiple patchy regions in the IT cortex of the macaque that selectively respond to face, color, and disparity (Komatsu et al. 1992; Tsao et al. 2006; Conway et al. 2007; Moeller et al. 2008; Harada et al. 2009; Lafer-Sousa and Conway 2013; Verhoef et al. 2015). These regions selective for either face or color are anatomically connected and form a neural network to process relevant features within the IT cortex (Moeller et al. 2008; Banno et al. 2010: see also Kravitz et al. 2013; Conway. 2018). With regard to gloss, it has been reported that in the marmoset, neurons strongly responsive to glossy stimuli are present in 2 regions of the IT cortex that are anatomically connected (Miyakawa et al. 2017; Miyakawa, personal communication). In the macaque, when an retrograde tracer was injected into a region of the STS where gloss-selective neurons were recorded, a dense cluster of labeled cells was observed in the TE region, anterior to the injection site, and in a more posterior region, around the TE/TEO border (Nishio et al. 2014, Annual Meeting of the Japan Neuroscience Society). In an functional magnetic resonance imaging (fMRI) study in the macaque (Okazawa et al. 2012), regions strongly responding to specular visual stimuli were observed around an area anterior to the posterior middle temporal sulcus (PMTS) and on the lower bank of the STS in the posterior part of IT (PIT). These areas roughly correspond to regions where strong connections were observed in the tracer experiment. Although no region sensitive to specular stimuli was observed in the anterior part of the IT (AIT) cortex in that fMRI study, that may be due to the reduction in BOLD signals in the AIT. We therefore hypothesize that there is a neural network connecting multiple regions within the IT cortex of the macaque that is related to the processing of gloss information. As we described in the previous paragraph, electrical stimulation likely activated the axons first. Electrical stimulation may have evoked activity in a bundle of efferent (feedforward) and/or afferent (feedback) fibers connecting the gloss-selective region examined in this study and other regions possibly related to the processing of gloss information. It has been shown that AIT neurons exhibit activities that are closely associated with the discrimination or categorization of visual stimuli (Jagadeesh et al. 2001; Baker et al. 2002; Sigala and Logothetis 2002). It seems reasonable to suggest that a region in the gloss processing network located more anteriorly is closely related to the gloss discrimination behavior. If so, anterior bias of the stimulation effect will be observed.

Another possible cause of the anterior bias that is not exclusive to the one just discussed is that the bias reflects the distribution of neurons selective for less glossy or matte stimuli. If these “matte” neurons tended to distribute more posteriorly than the neurons preferring higher gloss stimuli (high gloss-selective neurons) in the recorded region of the cortex, electrical stimulation of the posterior part could more strongly activate axons that feed into the matte neurons, and the activities of the dominant high gloss-selective neurons may be canceled out. This would result in high gloss-selective neurons having less effect on behavior. By contrast, when electrical stimulation was applied to the more anterior part, axons that feed into the gloss-selective neurons would be more strongly activated. A large majority of gloss-selective neurons are high gloss-selective and prefer higher gloss stimuli, and only a small number of matte neurons would be recorded (Fig. 2*A*, blue symbols). However, we should point out that the proportion of matte neurons may be higher. As we mentioned in an earlier paper (Nishio et al. 2012), our criteria for identification of gloss-selective neurons is conservative and may have underestimated the proportion of matte neurons. We identify a neuron as gloss selective only when its response does not show significant correlation between the optimal shape and the shuffled stimuli in which pixels are rearranged inside the border of the object (e.g., Supplementary Fig. 3). However, in the case of low gloss or matte stimuli, shuffling pixels does not cause a large change in the image, and the responses to the shuffled stimuli could easily correlate with responses to the original stimuli if a neuron preferred low gloss stimuli (i.e., matte neurons). Those neurons tended to distribute in a relatively posterior part of the recorded region, particularly the posterior–lateral part of the cortex (unpublished observation). Furthermore, in the present experiment, electrical stimulation at 3 sites in the posterior–lateral region in monkey T induced a behavioral shift to less glossy judgment (Fig. 3*C*,*b*). These results are consistent with the idea that one possible cause of the anterior bias of the stimulation effects may be the biased distribution of matte neurons.

### Muscimol Injection and Adaptive Regulation of Network Activity

In the muscimol injection experiment, a significant reduction in the sensitivity of gloss discrimination was observed after injection into the site where gloss-selective units were recorded in monkey G. That effect was larger 18 h after injection than after 30 min, and it was diminished at 42 h. Although muscimol inhibits neural activity in the vicinity of its injection site immediately after injection (Wardak et al. 2004; Liu and Snyder 2010; Van Dromme et al. 2016; Zhou and Freedman 2019), a previous report showed that the effect was maximum 18 h after injection (Chowdhry and DeAngelis 2008). Our observation is consistent with that report. The greater effect at the later time can be understood as the result of neural activity being inhibited not only in the immediate vicinity of the injection site but also across a larger area. It has been suggested that 18 h after injection, the muscimol has spread across a region with a diameter of 1–2 mm, and inhibition of neural activity occurs within a region about 3 mm in diameter (Martin 1991; Arikan et al. 2002). This is similar to the size of the region where the gloss-selective neurons were localized in the present experiment. In both hemispheres, there was a small region (gloss-selective region) in the cortex where the coordinates at which gloss-selective MUAs were recorded adjoin one another (Fig. 2*A*, (L21, A10), (L22, A10), (L21.5, A10.5) in monkey G, (L23, A13), (L24, A13), (L25, A13) in monkey T). It is therefore plausible that the muscimol-induced sensitivity reduction reflected the inhibition of the entire gloss-selective region. Effects of all experiments of muscimol injection in each monkey are summarized in Figure 6*C*,*D*. Interestingly, when we injected muscimol multiple times into the same gloss-selective region, the effect was greatest after the first injection and degraded with repeated injections. This suggests that neural activities in the targeted gloss-selective region did not contribute to gloss discrimination behavior after the second injection and that other cortical regions compensated for this. In the IT cortex, the gloss-selective region has been identified at similar positions in both hemispheres (Nisiho et al. 2012). Therefore, when neural activities in the gloss-selective region in one hemisphere is suppressed by muscimol injection, the counterpart in the opposite hemisphere can substitute. Furthermore, as we discussed above, a neural network consisting of multiple gloss-selective regions presumably exists in each hemisphere of the IT cortex. It has been reported that after muscimol injection into the lateral intraparietal cortex of a monkey performing a spatial attention task, whole brain fMRI showed increased activity in regions contained within the attention-related network, such as the frontal eye field. This suggests an adaptive change in the network after local suppression of the network (Balan et al. 2019). We would speculate that, in the present study, the monkeys’ gloss discrimination behavior relied on the activity of the entire neural network consisting of multiple gloss-selective regions and that the gain of each node (gloss-selective region) in the network is regulated to perform the discrimination optimally. We can think that after the muscimol injection inhibited the activity of the targeted gloss-selective region, the entire gloss-selective network adapted to the change by lowering the gain of the relevant node to maintain better performance. In one monkey (monkey T), the same tendency was observed and the sensitivity reduction was largest, though not significant, after the first muscimol injection into the gloss-selective region, and the effect decreased after repeated injections (Fig. 6*D*). To summarize, the results of the present study can be understood by considering that the gloss-selective region in the CIT recorded in our experiment is part of a larger gloss-selective network within the IT cortex and that gloss discrimination behavior reflects the functioning of the entire network.

### Possible Operation of a Gloss-Selective Network in the IT Cortex

It has been proposed that 2 characteristics of the neuronal population in the IT cortex that are important for visual object discrimination are sparseness and clustering (Op de Beek and Baker 2010). Feature-selective network organization likely adheres to that principle. At present, the existence of a gloss-selective network is speculative and mainly based on anatomical and fMRI experiments. It has not yet been determined whether neurons in the regions belonging to the hypothetical gloss processing network are selective for glossiness. Whether a gloss-selective region exists in the PIT and AIT and, if so, the kind of information that is expressed in those areas and how it differs from the gloss-selective region in the CIT are important issues to be addressed in the future. In an area of the PIT, anterior to the PMTS, which roughly corresponds to one of the nodes of the gloss processing network, the existence of neurons selective for a specific direction of luminance gradient or luminance contrast has been reported (Fujita et al. 1992; Komatsu et al. 2007). Luminance contrast is an important feature related to gloss perception (Ferwerda et al. 2001), and this information may be sent to the gloss-selective region in the CIT to generate gloss selectivity. With regard to the AIT, in addition to its role in the discrimination and categorization of visual stimuli, the AIT is thought to be involved in task-dependent control of cognitive function through top-down signals from other areas, including the prefrontal cortex. For example, it has been shown that color-selective neural activities in the AIT depend on the on-going task demand (Koida and Komatsu 2007). As a gloss signal can be used for many tasks, including object recognition, judgment of the condition of an object (e.g., freshness), and manipulation of objects, it will be important and interesting to investigate how gloss information is used in the context of task demands.

## Notes

The authors would like to thank M. Takagi and T. Ota for technical assistance with monkey treatment and the whole experiment; T. Shimokawa for providing three-dimensional shapes; N. Takahashi and I. Yokoi for assistance with the muscimol injection experiments. The authors also would like to thank M. Smith and R.H. Wurtz for providing manuals for muscimol injection techniques.

## Funding

This work was supported by the Japan Society for the Promotion of Science grant numbers 22135007 and 15H05916 (Grant-in-Aid for Scientific Research on Innovative Areas “Shitsukan” and “Innovative SHITSUKAN Science and Technology”) and 20H05955 (grant-in-aid for Transformative Research Areas (A) “Deep Shitsukan”) to H.K., 16K21587 (grant-in-aid for Young Scientists (B)) to A.N., and grant-in-aid for Research Activity start-up (19K24367 to M.B.).

## Supplementary Material

Gloss_perception_paper_CCC_supplement_2021-1-14_tgab011
