# Supplementary material for: Relationship Between the Activities of Gloss-Selective Neurons in the Macaque Inferior Temporal Cortex and the Gloss Discrimination Behavior of the Monkey
Source: Cereb Cortex Commun. 2021 Feb 10;2(1):tgab011. doi: 10.1093/texcom/tgab011 (PMC8152851; doi:10.1093/texcom/tgab011)
Supplement: Gloss_perception_paper_CCC_supplement_2021-1-14_tgab011 [file gloss_perception_paper_ccc_supplement_2021-1-14_tgab011.zip › Gloss_perception_paper_CCC_supplement_2021-1-14_tgab011.pdf]

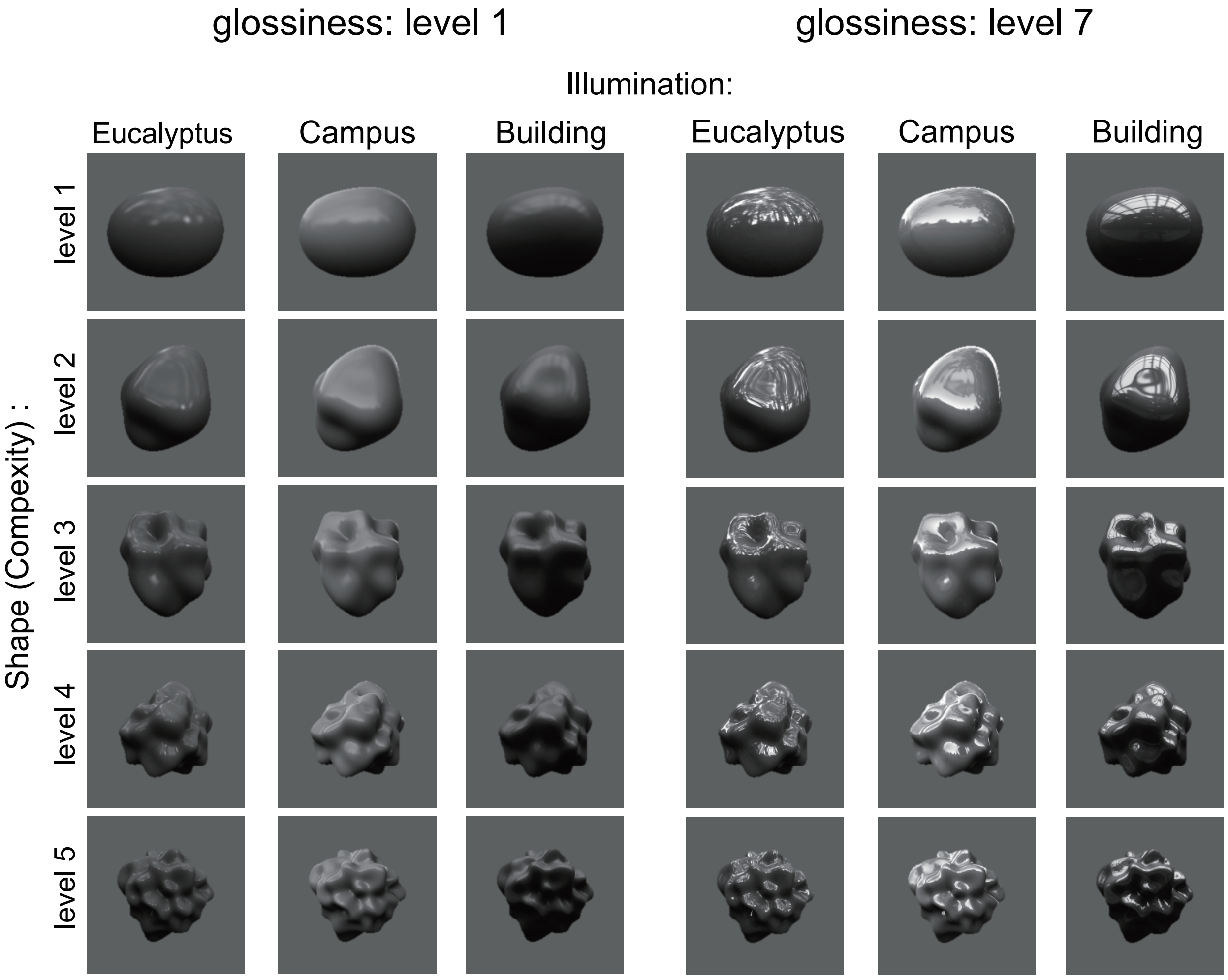

Figure-S1. Example images used in the gloss discrimination task.

Example images picked up from a stimulus set used for the gloss discrimination task in a daily experimental session. Of the seven levels of glossiness contained in the stimulus set, only the least glossy images (glossiness level 1) and most glossy images (glossiness level 7) are shown. For each glossiness level, stimuli consisted of a combination of object shapes with five levels of complexity (row) and three different illuminations (column). Different shapes were used for each daily session.

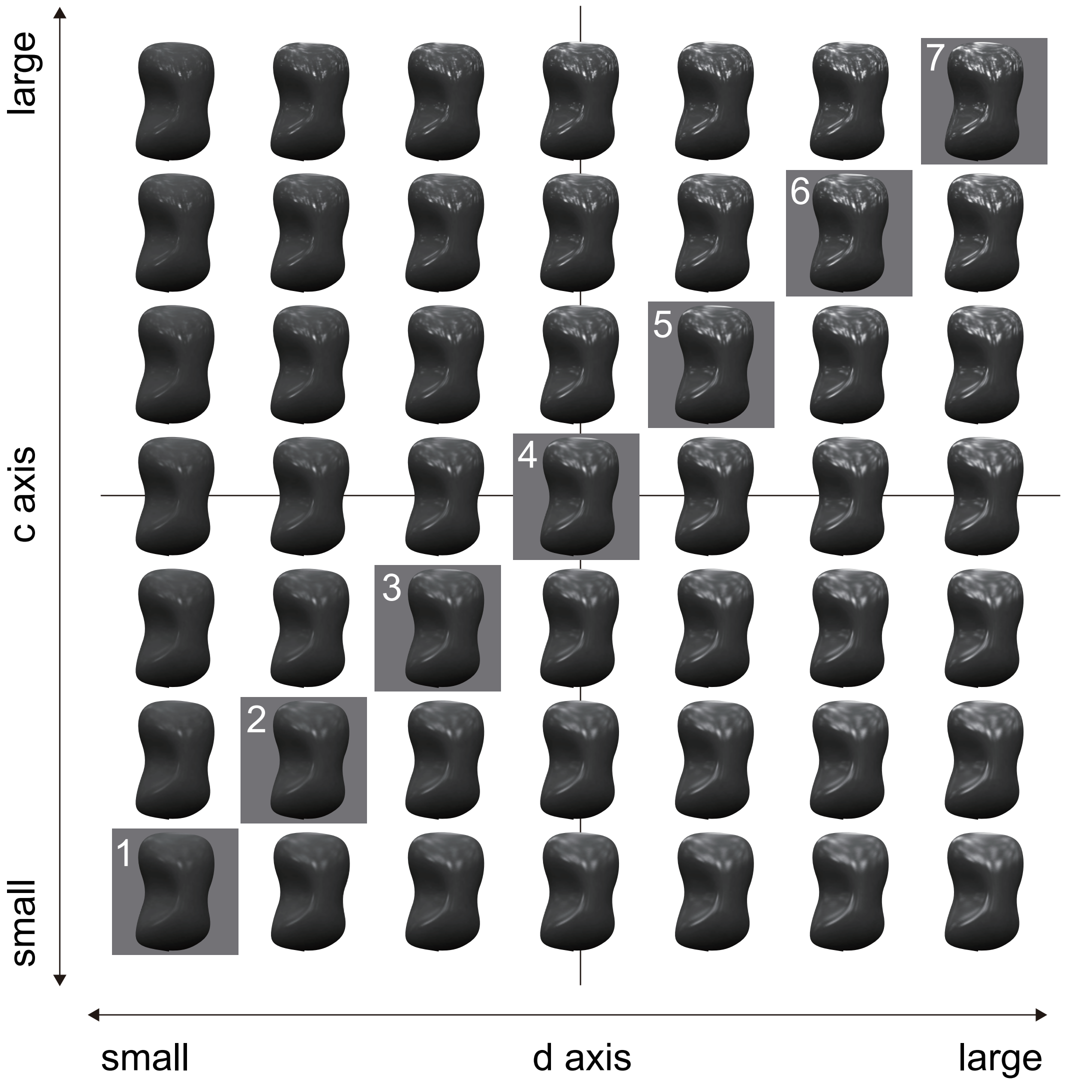

Figure-S2. Images of example objects having different combination of c and d parameters.

Parameter d changes at an equal step along the horizontal axis (d-axis) while parameter c changes at an equal step along the vertical axis (c-axis). The range of parameters are described in the main text. A total of 49 object images (seven c parameters x seven d parameters) are illustrated. Seven images along the diagonal line having gray background and inset numbers are examples of images used as the stimuli in the experiments. Both c and d parameters changed simultaneously in such a series of stimuli.

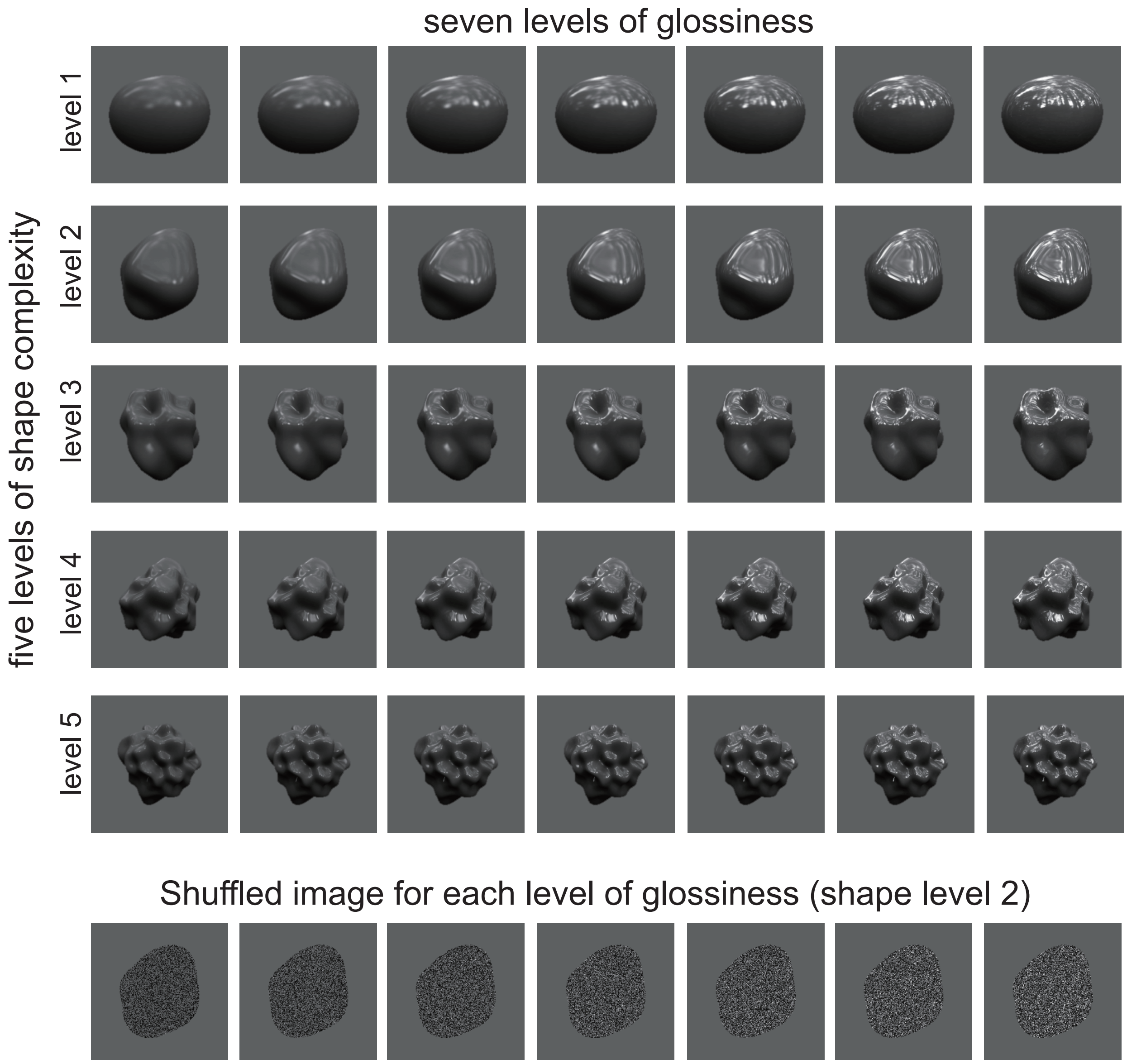

Figure-S3. Example images used in the neural selectivity mapping.

An example of the stimulus set used for testing neural selectivity for gloss. The stimulus set includes 35 images with a combination of seven levels of glossiness (row) and five levels of shape complexity (column). The illumination used in this set is Eucalyptus. The stimulus set also includes a set of shuffled images of objects. At the bottom, an example of a set of shuffled images of shape complexity level 2 is shown.

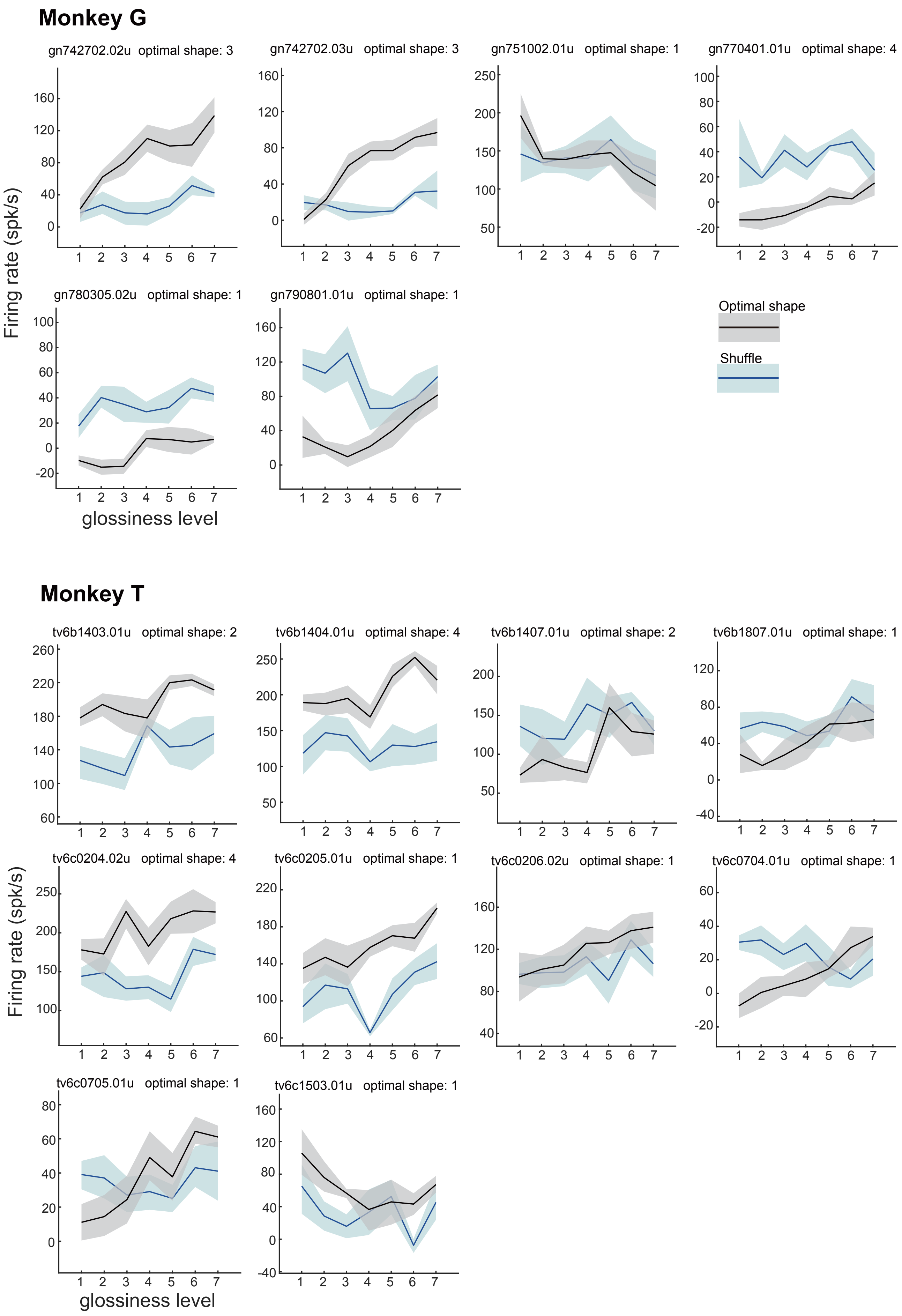

Figure-S4. Responses of all MUAs classified as gloss selective (n=16, 6 for monkey G, 10 for monkey T).

Responses to the optimal shape are indicated by black line and those to the shuffled images are shown by blue line. Error shadings indicate 95% confidence interval of the average (bootstrap test).

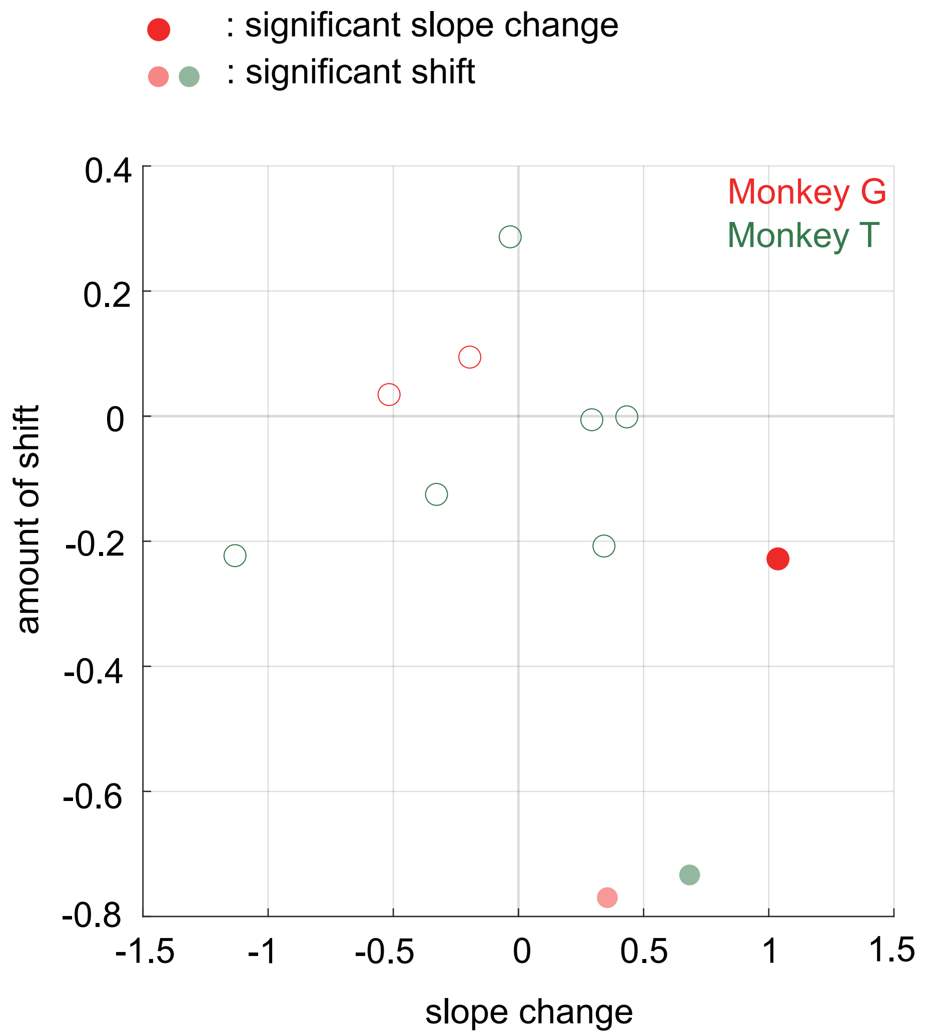

Figure-S5. Summary of the effects of muscimol injection.

Changes in the slope (horizontal axis) and the horizontal shift (vertical axis) of the psychometric function at 18 h after muscimol injection compared with those before injection are plotted for all the muscimol injection experiments (n=11). Color represents monkeys (red for G, green for T). Filled symbol represents significant effect ($p<0.05$, permutation test), and open symbol non-significant effect.

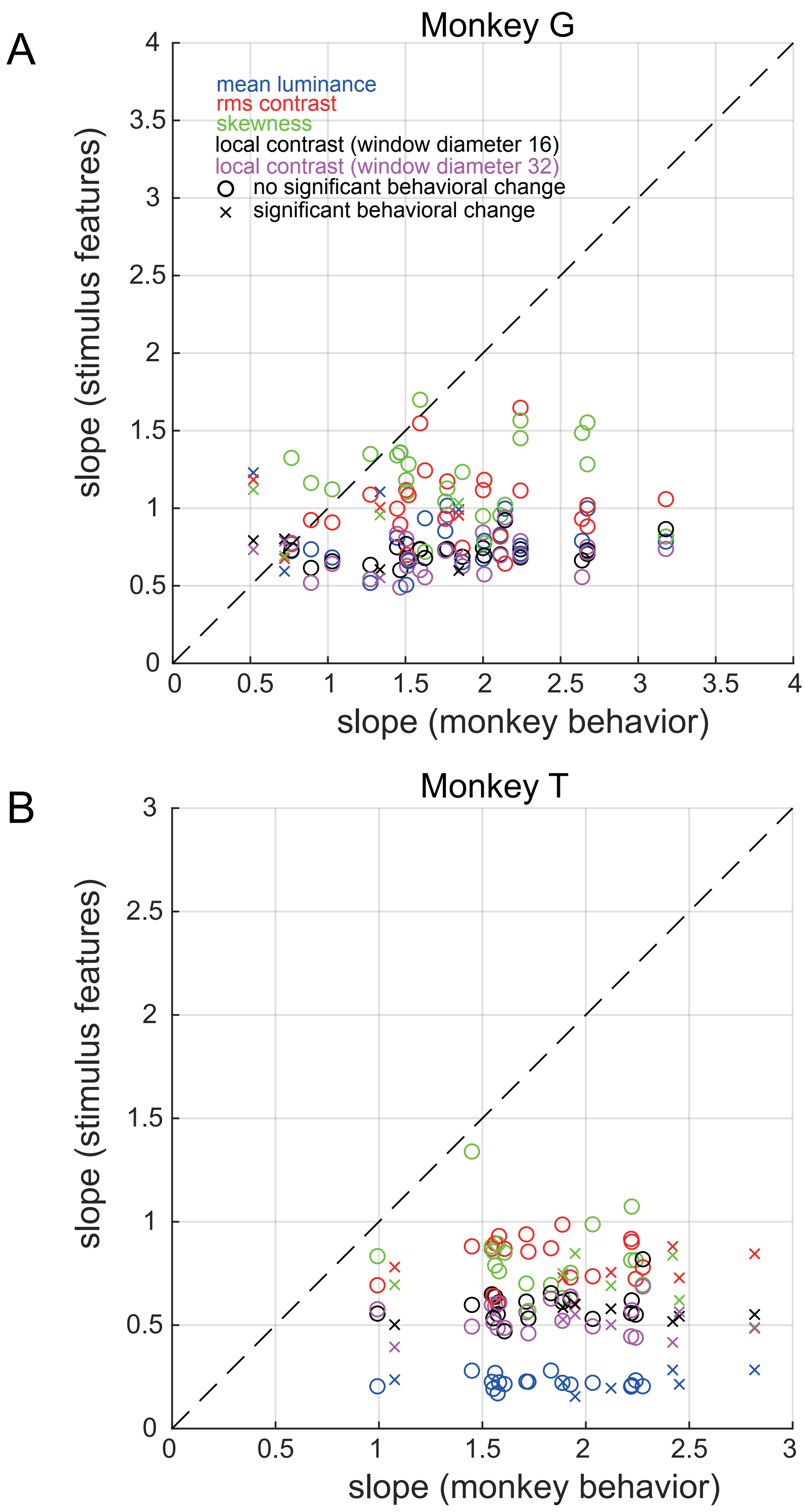

Figure-S6. Comparison of task performance of the monkey and that computed by ROC analysis based on stimulus features.

A: A comparison of task performance of monkey G (horizontal axis) and that computed by ROC analysis based on the stimulus features (vertical axis).

Different color of the symbol represents image features; blue: mean luminance, red: rms contrast, green: skewness of the luminance histogram, black: local contrast using 16 pixels patch diameter, and pink: local contrast using 32 pixels path diameter. Cross represents the experiment in which a significant behavioral change was induced by electrical micro-stimulation, and circle indicates the experiment in which no significant change was observed. Each symbol corresponds to one experimental session, and there are 140 symbols (28 sessions x 5 features).

B: A comparison of task performance of monkey T and that computed by ROC analysis based on the stimulus features. The format is same as in A. there are 125 symbols (25 sessions x 5 features).

Comparison between the performance of gloss discrimination behavior of the monkey (horizontal axis) and the performance computed by ROC analysis based on stimulus image features (vertical axis). For the latter, we used mean luminance, rms contrast, skewness of luminance histogram, and local contrast as the image features. The results for monkey G and T are shown in A and B, respectively. Pixel values inside the object contour were used for the computation. Local contrast was defined as in Mante et al. (2005). It was first computed within a local image patch (circularly raised cosine window) with one of two sizes (16 or 32 pixels in diameter), then the values were averaged across the region inside the object contour. In the ROC analysis, we first constructed the distribution of the values for a given image feature (e.g. mean luminance or rms contrast) for objects used in each daily session for each glossiness level. Secondly, the overlap of the distributions was quantified as an ROC curve between the images of glossiness level 4 (glossiness of the reference stimulus) and each of the seven levels of glossiness. There were 15 object images (five shapes x three illuminations) in each daily session. For monkey T, all 15 images were combined for the above computation, whereas for monkey G, the above computation was done separately for each illumination and then averaged because the illumination of the reference stimulus was the same as the target stimulus. Thirdly, psychometric function was generated by calculating the square measure of area under the ROC curve for each of seven glossiness levels. Finally, the slope of the psychometric function based on the image feature was compared with that obtained from the psychometric function based on the task performance of the monkey in the same daily session. Computation of local contrast was made using other sizes, and the results were basically the same.

Reference

Mante V, Frazor RA, Bonin V, Geisler WS, Carandini M. 2005. Independence of luminance and contrast in natural scenes and in the early visual system. Nat Neurosci 8:1690-1697

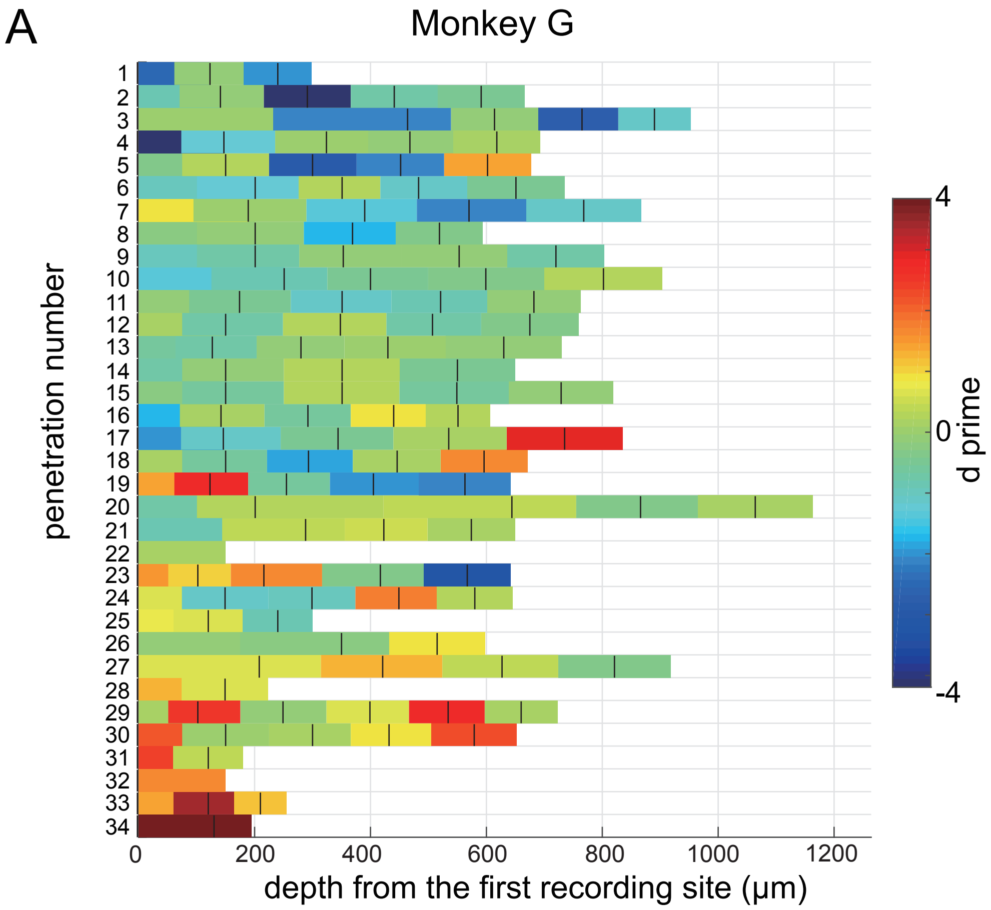

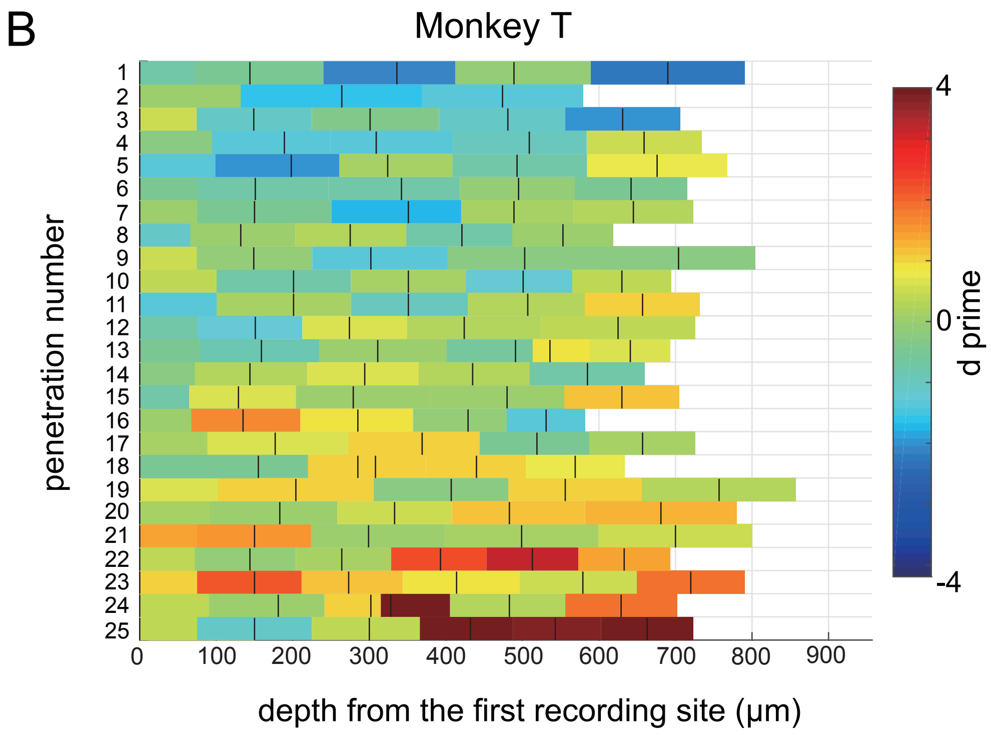

Figure-S7. Gloss selectivity of MUA versus relative cortical position.

Distribution of the gloss selective neurons in the depth direction are visualized in each penetration. The magnitude of the gloss selectivity of each MU recorded was quantified and color-coded, and plotted at the corresponding depth in each penetration in the same format as the data analyzed for the distribution of three-dimensional shape selectivity by Verhoef et al (Fig. S3 of Verhoef et al., 2012).

A: Distribution of the gloss selective units in monkey G. Horizontal axis indicates distance from the position of the first recording site in each electrode penetration, and vertical axis shows penetration number aligned in the ascending order of the mean d’ value. Color represents the value of d’ calculated by the difference between the responses to the least glossy stimulus (level 1) and those to the most glossy stimulus (level 7) among the stimulus set. Short black line in the middle of each colored region indicates the exact recording depth of each unit. When d’ was larger than +4 or smaller than -4, colors for +4 and -4 were used, respectively.

B: Distribution of the gloss selective units in monkey T. The format is the same as in panel A.
